# Ophthalmology and Artificial Intelligence: Present or Future? A Diabetic Retinopathy Screening Perspective of the Pursuit for Fairness

**DOI:** 10.3389/fopht.2022.898181

**Published:** 2022-05-10

**Authors:** Luis Filipe Nakayama, Lucas Zago Ribeiro, Fernando Korn Malerbi, Caio Vinicius Saito Regatieri

**Affiliations:** Retina and Vitreous Department, São Paulo Federal University (UNIFESP), Sao Paulo, Brazil

**Keywords:** artificial intelligence, ophthalmology, diabetic retinopathy, dataset, fairness

## Introduction

Computers that simulate human thought were first described in 1950, with the first artificial intelligence (AI) publication in 1943 describing a computer model that generated an autonomous binary output inspired by a human neuron (1). Since then, advances in machine learning and deep learning have expanded AI and created new paradigms in computer science. In healthcare, automatic processes facilitate diagnosis prediction, medical care, smart medical devices implementation, workflow improvement, electronic medical records interpretation, and screening programs (2, 3).

Convolutional neural networks are examples of deep learning analytics applied to image processing; they simulate interconnected neurons and provide output after multiple interconnected information layers (2). Machine learning algorithms could be unsupervised, supervised, or reinforced; in every learning method, the construction of datasets is a critical step (3).

More than twenty thousand articles have been published regarding AI in the last five years, with more than 1000 pertaining to ophthalmology. Regarding the retina subspecialty, AI has been applied in disease screening for diabetic retinopathy (DR), age-related macular degeneration (AMD), and retinopathy of prematurity (4). AI has already been applied in the IDx-DR system, the first FDA-approved device, with good results in Caucasian, North African, and Sub-Saharan populations (5). In the European Union, EyeArt has been used to exclude low-quality images, estimate DR progression, and recommend referral (5). Other algorithms, including Google’s, Singapore’s SERI-NUS, the Bosch DR Algorithm, and Retinalyze, have been developed (5). Algorithms have also been developed to support decision-making, e.g., in the anti-angiogenic treatment of AMD (6).

Despite technological advances, many challenges hamper real-world implementation of AI, such as variability in algorithm performance, patient acceptance in automated processes, and ethical conflicts. Therefore, this article’s objective was to compare characteristics of open-access retinal fundus photos datasets, implementations of AI in ophthalmology, and challenges to AI application in ophthalmology.

## Subsections

### Public Datasets Characteristics

Regarding open access ophthalmological public datasets of retinal fundus photos, the eyePACS is a public dataset from the US that captures images using a non-mydriatic Canon CR-DG1 or Canon CR1 camera in a three-image strategy: primary field image, disk-centered image, and temporal field. It comprises 88,702 images graded according to the International Clinical Diabetic Retinopathy (ICDR) DR classification. In the eyePACS labels, there is a description of patient sex, age, quality control, social aspects, or ethnicity (7).

The ODIR is a Chinese public dataset and uses images from Canon, Zeiss, and Kowa retinal cameras; it comprises 8,000 retinal images, classified as normal or regarding the presence of diabetic retinopathy, glaucoma, cataract, AMD, hypertension, myopia, and other conditions. The ICDR classification is applied in DR grading. In the ODIR labels, there is a description of patient age, but no description of sex, quality control, socioeconomic aspects, or ethnicity (8).

The APTOS is a public Indian dataset, composed of 5,590 retinal images. The fundus camera is not specified. In APTOS, ICDR diabetes grading criteria are applied. There are no descriptions of sex, age, quality control, socioeconomic aspects, or ethnicity in the APTOS labels [“(9) Blindness Detection” n.d.].

The DR1 and 2 are Brazilian public datasets of DR patients, composed of 1,597 images; the TRC-CW8 with a D90 Nikon camera was used. This dataset does not apply a specific DR grading scale and directly identifies retinal findings. In the DR1 and DR2 labels, there are no descriptions of sex, age, quality control, socioeconomic aspects, or ethnicity (10).

The IDRiD is another Indian public dataset comprising 516 images. A Kowa VX-10 alpha digital camera was used, and the ICDR DR grading scale was applied. In the IDRiD labels, there are no descriptions of sex, age, quality control, socioeconomic aspects, or ethnicity (11).

The Jichi is a Japanese public dataset composed of 9,939 images using an AFC-230 Nidek Fundus Camera. This dataset applies a modified Davis DR classification. In the Jichi labels, there are no descriptions of sex, age, quality control, socioeconomic aspects, or ethnicity (12).

The Rotterdam Ophthalmic Data Repository (ROD REP) is a public dataset from the Netherlands. This dataset is composed of 1,120 images; in a TRC-NW65 non-mydriatic Topcon digital fundus camera was used. The dataset evaluates intra and inter-visit registrations from 70 patients with diabetes. There are descriptions of sex and age in the ROD REP labels but no descriptions of quality control, socioeconomic aspects, or ethnicity (13).

The Methods to Evaluate Segmentation and Indexing Techniques in Retinal Ophthalmology (MESSIDOR 2) is a public dataset from France. This dataset includes 1,748 images using a TRC-NW65 non-mydriatic Topcon digital fundus camera and applies the ICDR as the DR grading scale. In the MESSIDOR 2 labels, there is a description of examination quality control but no descriptions of sex, age, socioeconomic aspects, or ethnicity (14).

The Tsukazaki is a public open-access Japanese dataset composed of 13,047 images; a 200Tx Ultrawide Optos camera was used. This dataset does not apply a specific DR grading scale. In the Tsukazaki labels, there are descriptions of sex and age but no descriptions of quality control, socioeconomic aspects, or ethnicity (15).

The Pathologic Myopia Challenge (PALM) is an open-access Chinese dataset composed of 1,200 images from a Zeiss Visucam camera. This is a pathological myopia dataset that does not apply DR classification distinction and classification. There is a description of patient age in the PALMS labels, and there are no descriptions of sex, quality control, socioeconomic aspects, or ethnicity (16).

Comprising the abovementioned datasets, there were a total of 131,459 assembled images, representing approximately 0.01% of the global population. However, the precise patient number is not always apparent in the datasets. The public datasets represent samples from the US, China, India, Brazil, Japan, the Netherlands, and France, with 188 non-represented countries. There is no dataset from low-income countries, four from middle-income countries (16,903 images), and five from high-income countries (112,808 images).

The ICDR was the most applied classification (five datasets with 104,556 images; 79.53% of the total). The sex of patients was described in two datasets (14,167 images; 10.78% of total), age in three datasets (22,167 images; 16.86% of the total), and quality control in one dataset (1748 images; 1.33% of the total). Socioeconomic aspects and ethnicity were not included in the labels of any dataset (**Table 1**). We excluded DIARETDB0, DIARETDB1, E-ophtha, UoA-DR from the analysis for containing fewer than 500 images.

**Table 1:** Comparison of features in ophthalmologic datasets.

DATASET	EYEPACS	ODIR	APTOS	DR 1 and 2	IDRiD	Jichi	ROD Rep	Messidor 2	Tsukazaki	PALM
**Images**	88702	8000	5590	1597	516	9939	1120	1748	13047	1200
**Country**	USA	China	India	Brazil	India	Japan	Netherland	France	Japan	China
**Grading**	ICDR	ICDR	ICDR	None	ICDR	Mod Davis	Non specified	ICDR	None	Non-applicable
**Sex**	No	No	No	No	No	No	Yes	No	Yes	No
**Age**	No	Yes	No	No	No	No	Yes	No	Yes	No
**Quality control**	No	No	No	No	No	No	No	Yes	No	No
**Social**	No	No	No	No	No	No	No	No	No	No
**Ethnicity**	No	No	No	No	No	No	No	No	No	No

### Commercially Available Diabetic Retinopathy Screening AI Platforms

IDx-DR was the first FDA-approved software package for DR screening in non-ophthalmic healthcare practice. It is a cloud-based software with a built-in retinal camera (TRC-NW400, Topcon) implemented in American institutions (5, 17). It evaluates image quality and detects retinal findings related to DR, providing classification regarding the referable disease. The IDx-DR reports a sensitivity of 96.8% for referable DR and a specificity of 59.4% (18).

RetmarkerDR is a DR screening software package developed and implemented by the Portugal DR screening program since 2011. It classifies retinal exams as normal or abnormal, determines quality assessment, and compares disease progression over time with a sensitivity of 95% for referable DR and a specificity of 63.2% (5, 18). The software package is a class IIa medical device in Europe and is approved in Australia (18).

EyeArt (Eyenuk Inc., Los Angeles, CA) is an automatic cloud-based DR software package from the European Union, Canada, approved by the FDA in 2020. This software package evaluates image quality; it estimates DR progression and provides classification regarding retinopathy referral, with a sensitivity of 91.7% for DR screening and specificity of 94.7% (5, 18, 19).

iGradingM provides disease/no disease grading in DR with a sensitivity of 97.4-99.1% and specificity of 98.3-99.3%. (18) It is validated in an English population and has been applied in the Southampton Diabetic Eye Programme (18, 20).

In developing countries, there are no commercially implemented AI software packages.

### Diabetic Macular Edema

Diabetic macular edema is a macular thickening secondary to diabetes and an important cause of vision loss, characterized by hard exudate deposition in fundus exam and abnormal OCT findings (21).

Algorithms are focused on screening edema through retinal fundus photos (22), predicting Optical Coherence Tomography findings from fundus photographs with better performance than retinal specialists (21), guiding treatment, and edema identification in OCT exam (23–25).

Limitations in AI algorithms focused on diabetic macular edema are the quality of labels and datasets applied, lack of standards, cross-validation, and interpretability.

## Barriers to AI Application

### Economic Challenges

Cost-effectiveness is a challenge to AI applications, especially in low and middle-income countries. It is necessary to consider the direct costs of hardware equipment, AI software, integrating AI systems, examination costs, and indirect costs of the camera operator and logistics in opposition to direct ophthalmologist evaluation in a cost-effectiveness analysis. Long-term costs such as maintenance software/algorithm upgrades must also be considered (26). Economic limitations are barriers to AI implementation in developing countries; equipment/technology prices and limited internet access in remote areas limit AI’s real-world application.

For IDX-DR, the retinal camera costs approximately $15–22,000 USD with fixed charges of $25 per patient for screening (17, 26). By comparison, in the Brazilian public health system, for example, an ophthalmologist evaluation costs $1.81, a retinal fundus evaluation costs $4.38, and a retinal fundus photo costs $4.46 (27), for a deficit of more than -$18.00 considering only the fixed charge in comparison with an ophthalmologist evaluation with a retinal fundus exam.

More affordable technology access and retinal cameras are necessary to spread AI implementation worldwide.

### Patient Acceptance

Resistance in implementing technology-assisted systems is a concern, especially regarding the possibility of AI mistakes and false-negative results (28).

Technological acceptance is directly related to patient age, with older people not used to daily technological uses, such as email and online internet activities (29). Another factor that needs to be considered is socioeconomic status, with a gap in technology access and acceptance among minorities (29).

### Ethical Concerns

Ethics in health AI are fundamental to guide developers, stakeholders, users, and regulators, providing standards for avoiding harness, promoting well-being, ensuring fairness, and dealing with individual autonomy to make decisions about their lives (30).

Patient data privacy and security are the primary concerns surrounding AI data sharing, with laws that protect the individuals’ rights and create obligations for data controllers (30). The United States Health Insurance Portability and Accountability Act regulate data privacy and security rules and provide guidelines for data sharing, imposing severe penalties for violations (31).

Individual privacy is essential in data sharing, and lack of privacy could lead to personal harm, affect personal dignity, and make vulnerable to cyber-theft (30). Before sharing, data needs to be de-identified (i.e., removal of identifying details, data, and other potentially identifying elements). Concerns about re-identification need to be considered (31). This concern is essential, especially for retinal photographs, because of the unique retinal vascular patterns that remain unchanged throughout a patient’s life (32).

Accountability and responsibility are also key points in AI ethics principles. Human supervision warranty applied in AI processes assures the responsibility and mechanisms are necessary to promote accountability in cases of wrong AI decisions (30).

Model interpretability is also a key point in AI ethics that leads to a lack of trust in AI. The obscure model decision-making process contributes to a loss of confidence in the algorithm decision (33).

AI models need to be reproducible, permitting knowledge transfer, implementation, and cross-validation. Algorithms should be interchangeable to contribute to AI evolution (34).

The ethical human-computer interaction is also a concert in autonomous AI systems, with no clear ethical rules in this interaction (35).

### Technological Limitations

Deep learning and convolutional neural networks extend the depth of layers with better prediction performance than traditional ML algorithms (J.-G. 36).

Computational speed is a limitation in large-scale data analysis. A high amount of Graphics Processing Units (GPU) memory and velocity is necessary for CNN implementation (37, 38).

In CNN trained on small datasets, the algorithm performance could be poor in outside data due to overfitting (39). Possible solutions for overfitting are data augmentation (generating image translations and horizontal reflections and altering the intensities of the RGB channels) and dropout (set to zero the output of hidden neurons with a probability of 0.5) (37).

## Discussion

AI technology can revolutionize medical care *via* intelligent, cost-effective, precise diagnoses and screening (40, 41). In ophthalmology, the most advanced applications are implemented in DR screening algorithms. Nevertheless, although many algorithms have been developed, barriers hamper real-world applications. The variable performance [even in FDA-approved algorithms (42)], economic inequality in developing countries, technophobia in the application of AI systems in daily practice, and ethical concerns remain challenges for the implementation of AI (28). Multidisciplinary groups including medical doctors, computer engineers, data scientists, and informatics technologists are necessary for implementing AI from benchmark algorithms to ethical autonomous healthcare tools (35). Ethics in AI applications are fundamental to achieving secure, fair, interpretable, reproducible, and accountable data sharing and algorithms.

A meta-analysis from Wu et al. and a review from Tsiknakis et al. and Jeong et al. concluded that the performance of Machine learning algorithms demonstrates high diagnosis accuracy but occurs bias in data selection and lack of algorithms validation (43–45).

AI application in developing countries remains an even bigger problem. Algorithms have been trained and tested with a few ethnic populations, mainly North American, European, and Asian patients (42). Open-access retinal fundus color photographs are primarily concentrated in developed countries, with 172 without available datasets and no representations (46). In this study, only six countries are represented.

Sex and age are available in a minority of open-access retinal fundus datasets. Socioeconomic aspects and ethnicity characteristics have not been considered in any ophthalmological dataset, creating a critical social bias in these algorithms.

Technical limitations due to examination quality in datasets create a problem in algorithm training, performance, validation, and implementation (A. Y. 42). In most publicly available datasets, rigorous quality control methodology is missing, and consequently, a trained ophthalmologist is often necessary for quality control, even in AI automated or semi-automated machines.

Some commercially available algorithms have been adopted in clinical practice in the US and Europe. However, in developing countries, economic gaps present barriers to the implementation of such technology in daily practice. Smartphone-based hand-held devices are a more cost-effective alternative, and automatic algorithms for DR screening have been described (47). Smartphone ophthalmoscopy applied in screening is more affordable but presents heterogeneous results in AI models, with benefits in resource-constrained health care countries. More studies are necessary with betters standards and with AI models development and validation (48).

The worldwide eye care professionals distribution is irregular, with two-thirds of global ophthalmologists in thirteen countries (China, USA, India, Brazil, Russia, Germany, Italy, Egypt, France, Mexico, Spain, and Polan), with higher national income directly associated with higher ophthalmologists availability (49). The unequal availability of eye care professionals within countries is also reported with big sociodemographic discrepancies (50).

The construction of datasets is a milestone for Data is a critical step in the development of machine learning algorithms, including their representativeness to achieve fairness in AI applications. An example is the UK Biobank, a large dataset that includes ethnicity, health conditions, and socioeconomic information (51). Machine learning has revolutionized medical care; nevertheless, critical points need to be accessed to reduce bias in algorithms and democratize access to technology.

## Author Contributions

LN and LR contributed to project leading, data acquisition, analysis, and data interpretation. All authors contributed to article conception, draft, and final version approval.

## Conflict of Interest

The authors declare that the research was conducted in the absence of any commercial or financial relationships that could be construed as a potential conflict of interest.

## Publisher’s Note

All claims expressed in this article are solely those of the authors and do not necessarily represent those of their affiliated organizations, or those of the publisher, the editors and the reviewers. Any product that may be evaluated in this article, or claim that may be made by its manufacturer, is not guaranteed or endorsed by the publisher.
